# Magnetic Stirring Device for Limiting the Sedimentation of Cells inside Microfluidic Devices

**DOI:** 10.3390/s24155014

**Published:** 2024-08-02

**Authors:** Sebastian Cremaschini, Noemi Torriero, Chiara Maceri, Maria Poles, Sarah Cleve, Beatrice Crestani, Alessio Meggiolaro, Matteo Pierno, Giampaolo Mistura, Paola Brun, Davide Ferraro

**Affiliations:** 1 Department of Physics and Astronomy, University of Padua, 35131 Padua, Italy; 2 Department of Medicine, University of Verona, 37124 Verona, Italy; 3 Department of Molecular Medicine, University of Padua, 35121 Padua, Italy

**Keywords:** microfluidics, cell sedimentation, magnetic stirring, microfabrication, 3D printer

## Abstract

In experiments considering cell handling in microchannels, cell sedimentation in the storage container is a key problem because it affects the reproducibility of the experiments. Here, a simple and low-cost cell mixing device (CMD) is presented; the device is designed to prevent the sedimentation of cells in a syringe during their injection into a microfluidic channel. The CMD is based on a slider crank device made of 3D-printed parts that, combined with a permanent magnet, actuate a stir bar placed into the syringe containing the cells. By using A549 cell lines, the device is characterized in terms of cell viability (higher than 95%) in different mixing conditions, by varying the oscillation frequency and the overall mixing time. Then, a dedicated microfluidic experiment is designed to evaluate the injection frequency of the cells within a microfluidic chip. In the presence of the CMD, a higher number of cells are injected into the microfluidic chip with respect to the static conditions (2.5 times), proving that it contrasts cell sedimentation and allows accurate cell handling. For these reasons, the CMD can be useful in microfluidic experiments involving single-cell analysis.

## 1. Introduction

One of the main goals of microfluidics is the development of the so-called “lab-on-a-chip”, integrating the protocols commonly performed in large laboratories within small and portable devices [1,2,3]. Through the pursuit of this goal, over the past twenty years, several examples of microfluidic devices have been proposed to handle and analyze various biological samples, e.g., body fluids [4,5], nucleic acids [6,7], proteins [8], and cells [9,10]. In particular, the integration of single-cell analysis in microfluidic systems is currently revolutionizing cell biology, allowing the study of cellular heterogeneity in a high-throughput manner [11].

One of the most common limitations in the handling of cells within microfluidic devices is their sedimentation. In fact, most cells are denser than the surrounding aqueous phase and, under the influence of gravity, they tend to sediment within the container. In detail, the sedimentation speed v is driven by Stokes’ law $v=d^{2}\left(\rho_{o}-\rho_{f}\right)g/\left(18\;\mu_{f}\right)$, where d and $\rho_{o}$ are, respectively, the diameter and density of the cell, $\rho_{f}$ and $\mu_{f}$ are the density and viscosity of the surrounding liquid, and g is the acceleration of gravity [12,13]. For example, considering a cell of 10 µm ($\rho_{0}$ ~ 1 g/cm^3^) dispersed in water ($\rho_{f}\;\sim \;0.997$ g/cm^3^,${\;\mu }_{f}$ ~ 1 mPa∙s), its sedimentation speed is about 10 µm/min.

To avoid the problem of sedimentation, a conventional approach is to resuspend cells by gently pipetting the solution every few minutes [14]. However, this method cannot be applied in microfluidic experiments because of the small volume typically employed and the confinement of liquids in closed containers (e.g.,: syringes, tubes, or capillaries). Another approach is to match the density between cells and the surrounding medium [15,16]. However, this method can slow the sedimentation but cannot completely prevent it; additionally, the choice of the right density-matching reagent may influence the experiment [17].

To face these limitations, some strategies have been proposed in the literature that can be divided into (i) mechanical movement of the liquid container and (ii) magnetic stirring. For the first, rotating syringe pumps have been developed to continuously resuspend polystyrene beads [18]; however, this approach has not been tested using cells and requires a complex fabrication process. We recently proposed a method based on mechanical shaking at high frequency [19]. This method is effective for magnetic beads, but it could induce cell damage. Differently, the approaches exploiting magnetic stirring generally consist in adding a stir bar in the container that can be actuated by an external magnet [20,21,22]. However, these methods have been validated using polymeric microparticles, but without employing living cells. Well-known, simple, and low-cost approaches suggest collecting both the cell suspension and the stir bar in the same syringe and agitating a permanent magnet near the system [23,24]. However, no data are reported on mixing efficiency and cell viability. Similarly, these performances are not characterized by the commercial technology proposed for this scope (i.e., Nemix-50, by Cetoni, Korbussen, Germany).

Inspired by existing methods, in this work, we present an automated cell mixing device (CMD) that contrasts cell sedimentation in a syringe during a microfluidic experiment, validated in terms of cell viability and injection within a microchannel. In detail, the CMD consists of a slider crank mechanism, fabricated with 3D-printed components, devoted to transfer the circular motion of a gear motor to the linear movement of a permanent magnet, which is used to activate the motion of a stir bar placed inside a syringe. In particular, in this way, the stir bar is moved back and forth along the entire length of the syringe in an automated manner. After mechanical characterization, the device is characterized at two levels of complexity by using lung cancer cell lines (A549): at first, in terms of cell viability under various agitation conditions (i.e., by varying the oscillation frequency and mixing time), and then, by evaluating the injection frequency of cells within a microfluidic device. Interestingly, this approach shows a higher injection efficiency with respect to static conditions (about 2.5 times). Given this result, the proposed device can be easily integrated into all microfluidic experiments that consider constant and homogeneous cell injection in the microfluidic channel, such as single-cell analysis.

## 2. Materials and Methods

### 2.1. Creation of the Cell Mixing Device

**Figure 1:**
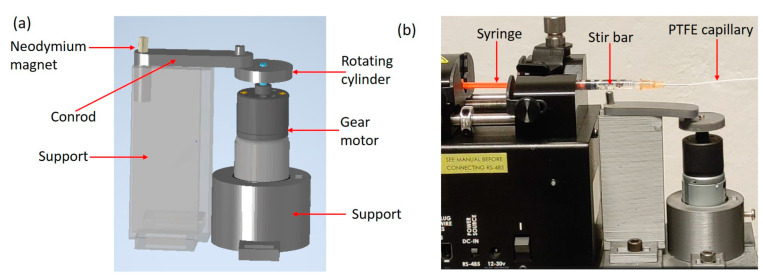
(**a**) Three-dimensional structure of the CMD. (**b**) Picture of the CMD moving the magnetic stir bar inside a plastic syringe, connected to the syringe pump. The PTFE capillary is connected to the inlet of the microfluidic chip for cell counting experiments.

The cell mixing device (CMD) shown in Figure 1a is based on a slider crank mechanism: a 12 V DC gear motor (Distrelec, HL149.12.43, Milan, Italy) is coupled to a rotating cylinder, which transfers its circular motion to a conrod embedding a cylindrical permanent magnet (S-05-08-N, Supermagnete, Gottmadingen, Germany). In this way, the circular motion of the gear motor leads to a linear back and forth motion of the magnet. The gear motor is connected to a power supply (Elind 32DP8, Metaf, Siena, Italy), which allows the voltage to be varied continuously between 0 and 12 V. The slider crank and holders are made by a stereolithographic 3D printer (Form 3, Formlabs, Somerville, MA, USA), using Grey V4 resin (by Formlabs, Somerville, MA, USA). The source files (in STL format) for the 3D-printed components can be found in the Supplementary Information section (see Note S1, Supplementary Materials).

As shown in Figure 1b, the cell mixing device is placed in correspondence to a 1 mL plastic syringe (plunger length 88 mm, internal diameter 4.43 mm, and volume 1 mL; Pikdare S.p.A., Como, Italy), containing a small magnetic stir bar (length 2 mm and diameter 2 mm; 442-0359, VWR International Srl, Milan, Italy) washed with ethanol, and mounted on a syringe pump (11 Elite Module Pico Single, Harvard Apparatus, Holliston, MA, USA). The syringe is connected to the device through a 25-gauge needle (Like Sun GmbH, Essen, Germany), PTFE capillaries (inner/outer diameter 0.5/1 mm; Sigma Aldrich, Darmstadt, Germany) and a silicone joint (inner/outer diameter 0.5/1 mm; Deutsch & Neumann, Henningsdorf, Germany). The syringe is mounted in a horizontal configuration as typically performed in microfluidic experiments involving cell injection into microchannels [16,25]. The syringes and needles used for the experiment were purchased in a sterile package and used immediately after opening.

When the motor is activated, the stir bar is continuously moved back and forth due to magnetic interaction with the permanent magnet placed in the conrod (see Video S1, Supplementary Materials).

### 2.2. Microfluidic Device Preparation

**Figure 2:**
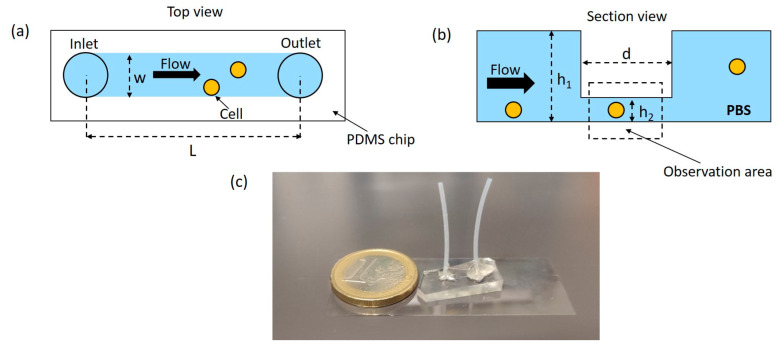
Schematic (**a**) top and (**b**) section views of the microfluidic chip used for cell counting (schemes are not scaled). The width w of the channel is 250 μm, and the height of the two layers h_1_ and h_2_ is 160 and 30 μm, respectively. The total length L is 9 mm, while the length of the thinnest part d of the chip is 1 mm. The latter corresponds to the observation area for counting the cells flowing in the microchannel within PBS. Light blue color indicates the microchannel, while orange circles represent the flowing cells. The white rectangle in (**a**) is the PDMS chip. Black arrows indicate the flow direction inside the microchannel, from the inlet to the outlet. (**c**) Picture of the final microfluidic chip made on PDMS and with PTFE capillaries fixed using epoxy glue.

The design of the microfluidic chip for cell counting is reported in Figure 2a,b: it presents a straight channel with rectangular cross section (height of 160 μm, width of 250 μm, and total length of L = 9 mm). In the center of the channel, the height is reduced to 30 μm over a length of d = 1 mm, to better observe and count cells flowing into the microfluidic chip. A deeper channel would prevent cells from passing out of the focal plane of the objective, whereas a channel with uniformly low height would lead to a higher pressure drop inside of the microchannel.

The microfluidic device for cell counting is prepared using a replica molding technique, starting from a master prepared using double-layer photolithography (see Figure 2c). In detail, two overlapping SU8-2050 layers are realized by spin-coating on a silica substrate, with thicknesses h_1_ = 30 μm and h_2_ = 160 μm. The photolithography process is performed using micro maskless-aligner technology (Heidelberg Instruments, Germany); the exposure doses are 220 and 300 mJ/cm^2^, while the percentages of defocusing are −40% and −60% for the thinner and larger layers, respectively. The master is coated with 1H,1H,2H,2H-Perfluorooctyltrichlorosilane (Alfa Aesar, Kandel, Germany) to create an anti-sticking layer and prevent adhesion of PDMS [26]. The microfluidic chip is made of polydimethylsiloxane (PDMS, Sylgard 184, Dow Corning, Midland, MI, USA) using conventional soft-lithography and bonded to a glass coverslip using oxygen plasma treatment [27]. PTFE capillaries are fixed in the inlet and outlet using epoxy glues (Bostik^®^, Bolton Adhesives, Rotterdam, Netherlands). Finally, the microchannel walls are treated with a solution of Phosphate-Buffer Saline (1X PBS) to which 0.01% w/v of Bovine Serum Albumine (BSA) is added; the latter prevents cell adhesion to the channel walls due to electrostatic interaction [28]. The final microfluidic chip is reported in Figure 2; it has a width (w) of 250 μm and a total length between the inlet and the outlet (L) of 9 mm, while the length of the thinner part of the channel (d) is 1 mm. The latter corresponds to the observation area for image acquisition during cell counting experiments. The tubing and microfluidic chips are not sterilized; new chips and tubing are used for experiments carried out on different days.

### 2.3. Optical Setup for the Characterization and Validation of the Cell Mixing Device

**Figure 3:**
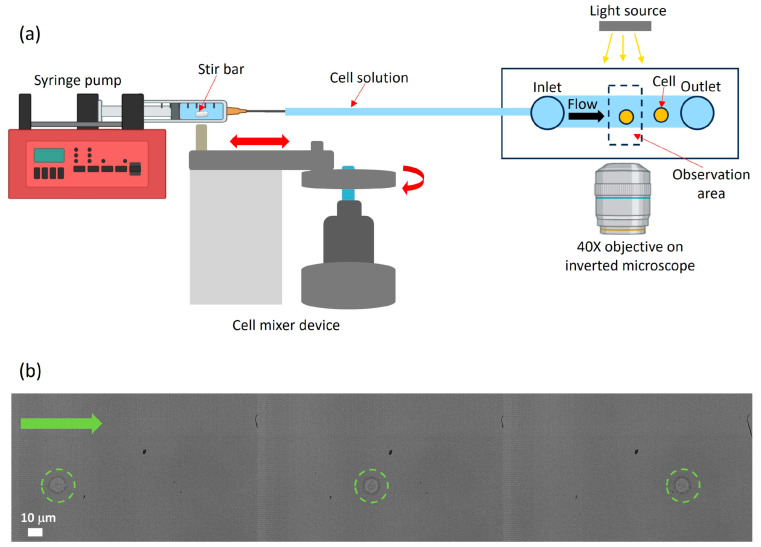
(**a**) Scheme of the experimental setup used for cell counting experiments. Black arrow indicates the flow direction inside the microchannel. (**b**) Image sequence of a cell (highlighted by green dotted circles) flowing within the microchannel from left to right; the green arrow indicates the flow direction.

The experimental setup for the acquisition of image sequences is shown in Figure 3a; a fast camera (VEO-E-310L, Phantom, Wayne, NJ, USA) is mounted on an inverted microscope (Eclipse Ti2, Nikon, Tokyo, Japan) equipped with a 40X objective (MRL00402, Nikon, Tokyo, Japan) and a white LED light (Nikon, Tokyo, Japan) for illumination. For cell counting experiments, cells are observed flowing in the thinnest part of the microfluidic channel (see Figure 3b). The cell number is quantified for three different time intervals from the start of the cell infusion: (i) between 5 and 20 min, (ii) between 35 and 55 min, and (iii) between 70 and 90 min. For each time interval, sequences of 18 s are acquired every minute at a frame rate of 200 fps (see Video S2 in the Supplementary Materials).

The analysis of the videos and the cell counting is performed by exploiting a combination between tools specific of ImageJ software 1.54j and a Labview 2024 program (see Note S2 and Figure S1, Supplementary Materials).

### 2.4. Cell Cultures

A human lung adenocarcinoma A549 cell line (CCL-185, ATCC, Manassas, VA, USA) is cultured in Dulbecco’s Modified Eagle Medium (DMEM, Gibco™, Fisher Scientific, Waltham, MA, USA, 41965-039) supplemented with 10% of Fetal Bovine Serum (FBS), 1% penicillin/streptomycin 100X. Cells are grown at 37 °C in a humified atmosphere and 5% CO_2_.

**Figure 4:**
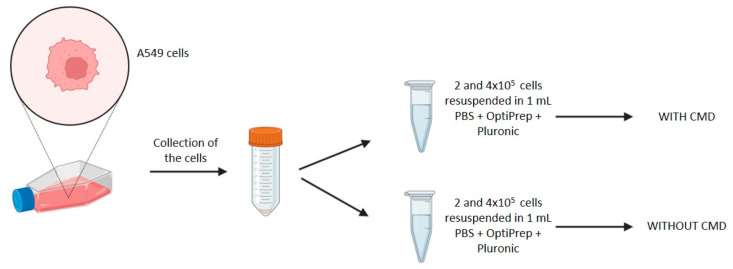
Workflow of cell preparation procedure: A549 cells are collected from the flask and centrifuged to obtain a pellet. The cells are then resuspended in 1 mL of PBS 1X solution, with 150 µL OptiPrep and 1 µL 10% Pluronic, for each experimental condition (with and without CMD).

For each experiment, 70–80% confluent cells are detached with trypsin and centrifuged at 1200 rpm for 5 min. The obtained pellet is then resuspended with an appropriate volume of fresh cell culture medium, and cell viability is quantified using a Trypan Blue exclusion assay. Specifically, a suspension of cells is mixed with Trypan Blue dye and loaded on the hemocytometer. The viable (unstained) and non-viable (stained) cells are visualized and counted under the microscope. Once the viability and cell number are verified, the cells are centrifuged at 1200 rpm for 5 min and resuspended in 1 mL of PBS 1X solution with 150 μL of OptiPrep™ Density Gradient Medium (Sigma Aldrich, St. Loius, MO, USA, D1556) and 1 μL of 10% Pluronic^®^ F-68 (Gibco™, Fisher Scientific, Waltham, MA, USA, 24040-032) (see Figure 4) [15].

## 3. Results

The performance of the CMD is evaluated by (i) characterizing its mechanical behavior, (ii) studying cell viability under different conditions, and (iii) monitoring the number of cells flowing inside the microchannel at different time intervals, with and without the CMD.

### 3.1. CMD Mechanical Characterization

**Figure 5:**
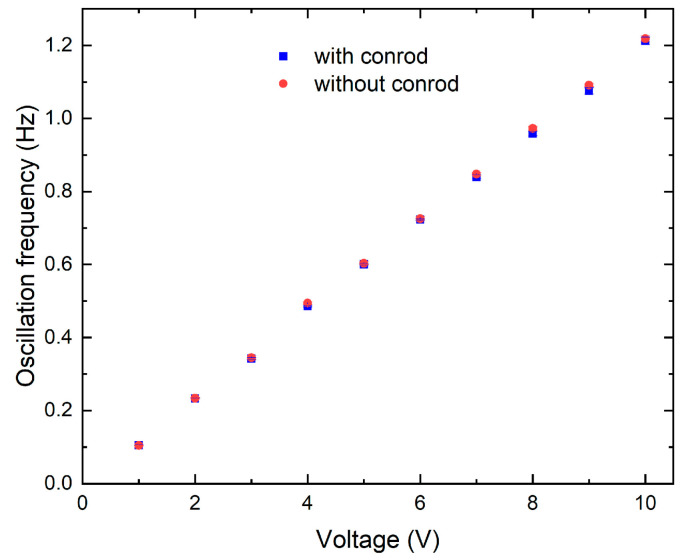
Frequency characterization of the cell mixing device (CMD), by varying the voltage applied to the gear motor with (blue square) and without (red circle) the conrod. If not visible, error bars are smaller than the data points.

As shown in Figure 5, the cell mixing device is first characterized by evaluating the oscillation frequency for different voltages (V) applied to the gear motor, under two different conditions: (i) by assembling the moving conrod on the CMD (with conrod), and (ii) by using only the rotating cylinder (without conrod). In the first case, the oscillation frequencies are determined by measuring the total time interval of 10 consecutive oscillations with a chronometer, and then the time for a single oscillation is computed. The oscillation frequency is determined as reciprocal of the time for a single oscillation. For each voltage applied, ten repeated measurements are performed; the final frequencies and related error bars are calculated as average values and standard deviations, respectively. In the second configuration (without conrod), the same procedure is repeated considering the rotations of the cylinder mounted on the gear motor. Figure 5 shows the values of the oscillation frequencies as a function of the voltage applied to the gear motor for both configurations. The oscillation frequency varies linearly by changing the applied voltage in both cases. In detail, by actuating the motor between 0 and 10 V, it is possible to move the magnet back and forth with a frequency up to 1.20 Hz. The two datasets overlap in the investigated range, showing that the components of the slider crank do not induce mechanical problems. If a less efficient motion conversion was observed, the power of the gear motor should be increased.

### 3.2. Cell Viability

**Figure 6:**
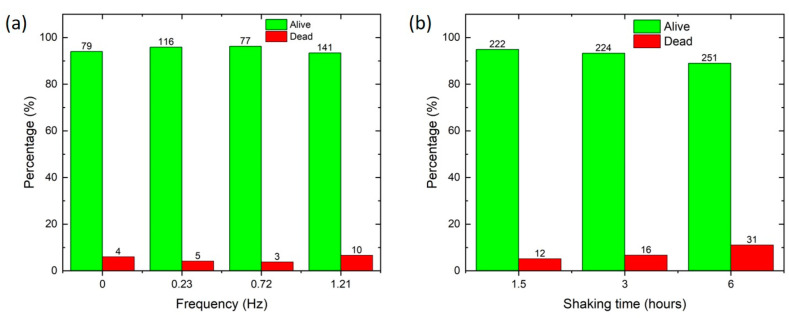
(**a**) Cell viability quantification by increasing oscillation frequency; each experiment is performed for 90 min. Data at 0 Hz are taken with the CMD not activated. (**b**) Cell viability for longer mixing times (between 1.5 and 6 h) at a frequency of 0.72 Hz (6 V). The number above each column corresponds to the total number of cells alive or dead counted in each experiment. The cell concentration in the syringe is 4 × 10^5^ cells/mL in both cases.

Cell viability is evaluated by counting the total number of alive and dead cells after a defined time (1.5, 3, and 6 h) and specific mixing conditions (between 0 and 1.2 Hz). Specifically, the syringe with the magnetic stir bar placed inside is prefilled with 500 μL of cell suspension; then, the cell mixing device is activated and after the chosen time, cells are extracted from the syringe and their viability is quantified. Cell viability is evaluated by varying the oscillation frequency of the conrod and the overall mixing time. Initially, before injecting into the syringe, the cell viability is found to be about 97%. Figure 6a shows that the mixing frequency applied to the cells does not affect their viability, which is about 95% for all investigated cases, including the case without mixing (0 Hz). This suggests that the CMD does not induce cell death. However, Figure 6b reports that by fixing the oscillation frequency at 0.72 Hz, cell viability decreases from about 95% after 1.5 h to 90% after 6 *h* of incubation in the syringe at room temperature.

Given this characterization, during subsequent experiments, the chosen parameters are 0.72 Hz (corresponding to 6 V) for the oscillation frequency and 1.5 h for the experimental time, respectively.

### 3.3. Cell Counting Experiments

To evaluate the applicability of the cell mixing device, cell counting measurements are performed by mounting the setup shown in Figure 2a: a plastic syringe is filled with approximately 500 μL of cell solution and then the solution is injected into the microfluidic chip with a computer-controlled syringe pump, setting a working flow rate of 250 μL/h. The connection between the plastic syringe and the inlet of the microfluidic chip is approximately 20 cm long and is as short as possible to prevent cell sedimentation within the PTFE capillary. This connection has a volume of about 40 μL.

**Figure 7:**
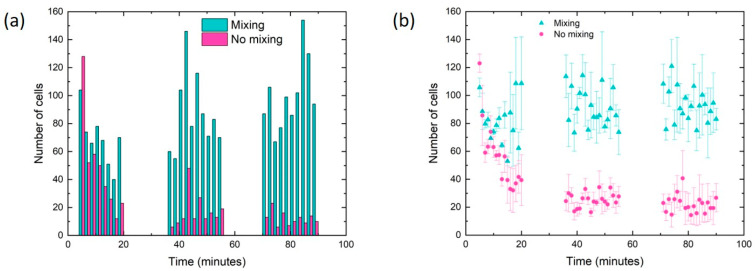
(**a**) Histogram showing the number of cells counted for each image sequence over 90 min of experiment, under both mixing and no mixing conditions for one specific experiment (*n* = 1). (**b**) Averaged data of cell number as a function of time, collected from experiments performed on different days (*n* = 3). In all experiments, the oscillation frequency is set to 0.72 Hz and the cell concentration is 2 *×* 10^5^ cells/mL.

Video acquisition and image analysis are performed with the procedure described in Section 2.3 and Note S2 of the Supplementary Materials. For each experiment, two different conditions are compared: in the presence (mixing) and absence (no mixing) of the CMD. Different and independent experiments are performed on three different days to ensure reproducibility in the behavior of the CMD. Figure 7a reports data from one of the three experiments, comparing mixing and no mixing conditions. The complete acquired data are reported in Figure S2 in Note S3 of the Supplementary Materials. Figure 7b, showing the averaged data acquired from all the experiments, clearly indicates that during the first 10 min, cell injection is similar for both mixing and no mixing conditions. Afterwards, the presence of the CMD allows keeping constant the cell injection during the entire investigated time range (90 min), while the injection rate dramatically decreases (by about 2.5 times) in the no mixing case due to the cell sedimentation inside the syringe.

## 4. Discussion and Conclusions

We have developed a cell mixing device (CMD) based on magnetic stirring and made with 3D-printed components. The device is tested for up to 6 h of continuous mixing without showing any variation in the mechanical behavior and does not require any lubricant.

The CMD functionality is characterized using lung cancer cells (A549 cell line). The cells have high viability under different mixing conditions, by varying both the oscillation frequency and the total mixing time. It is noteworthy that cell viability is always about 95% for the entire range of the oscillation frequency considered (up to 1.20 Hz). Moreover, Figure 7b shows that cell viability is 95% for experiments lasting up to 3 h, while if the total time duration reaches 6 h, cell viability decreases to 89%. Therefore, the mixing process is not responsible for cell mortality.

The performance of the CMD is then evaluated by monitoring the number of cells flowing inside a microfluidic chip at different time instants, showing the great importance of the CMD in reducing cell sedimentation. During the first 10 min of cell injection, the number of cells arriving in the microchannel is similar in both mixing and no mixing cases, whereas the difference between the two is remarkable afterwards: in the no mixing experiments, the number of incoming cells drastically reduces because of the cell sedimentation inside the syringe. On the other hand, by using the CMD, cell injection is constant over 90 min, which can be considered a typical time duration of a microfluidic experiment. Additionally, according with Figure 7, the cell mixing is not affected by the fact that the stir bar is confined in a progressively smaller volume of the syringe. These results are reproducible in different experiments carried out on different days.

A density gradient medium (i.e., OptiPrep™), which is typically employed to prevent cell sedimentation in microfluidic experiments, is added to the cell suspension [16,29,30,31]. However, the sedimentation process is not completely prevented, as shown in Figure 7. The gentle and constant agitation provided by the CMD allows improving the cell injection into the microchannel.

Our cell mixing device combines magnetic stirring and mechanical motion in a continuous and automated manner. Furthermore, compared to similar approaches [16,19,20,21,22], it has been quantitatively validated in terms of cell viability and efficacy in cell injection in a microfluidic channel. Then, being based on a simple gear motor and 3D-printed components, it represents a reliable and low-cost alternative to more complex and expensive technology. In fact, the price can be estimated to be about 50 euros. In case a larger sample volume is required with a greater syringe, it is recommended to employ a bigger size stir bar to take advantage of the mixing performance.

Therefore, the CMD can be safely implemented in any microfluidic experiment involving the presence of biological samples, such as single-cell sequencing or encapsulation in droplet experiments [11,25,32].

## Data Availability

Data are contained within the article or Supplementary Materials.

## Supplementary Materials

The following supporting information can be downloaded at: https://www.mdpi.com/article/10.3390/s24155014/s1; Video S1: Cell mixing device (CMD); Video S2: Cells flowing in the microfluidic channel; Note S1: Three-dimensional design of the cell mixing device (*.stl); Note S2: Image analysis procedure; Note S3: Cell counting data of the experiments performed on different days.
