# Supplementary material for: Magnetic Stirring Device for Limiting the Sedimentation of Cells inside Microfluidic Devices
Source: Sensors (Basel). 2024 Aug 2;24(15):5014. doi: 10.3390/s24155014 (PMC11314744; doi:10.3390/s24155014)
Supplement: Supplementary file 1 [file sensors-24-05014-s001.zip › Supplementary information_proof.pdf]

Supplementary information

Magnetic Stirring Device for Limiting the Sedimentation of Cells inside Microfluidic Devices

Sebastian Cremaschini [1], Noemi Torriero [1], Chiara Maceri [1], Maria Poles [2], Sarah Cleve [1], Beatrice Crestani [1], Alessio Meggiolaro [1], Matteo Pierno [1], Giampaolo Mistura [1], Paola Brun [3] and Davide Ferraro [1*]

[1] Department of Physics and Astronomy, University of Padua, 35131 Padua, Italy;
[2] Department of Medicine, University of Verona, 37124 Verona, Italy;
[3] Department of Molecular Medicine, University of Padua, 35121 Padua, Italy;
* Correspondence: davide.ferraro@unipd.it

Video S1: Cell mixing device (CMD)

Video of the cell mixing device (CMD) actuating the back-and-forth movement of the prefilled magnetic stir bar in the syringe with cell suspension. The CMD is moved with a frequency of 0.72 Hz, corresponding to 6 V applied to the gear motor.

Video S2: Cells flowing inside microfluidic channel

Video of cells flowing through the microfluidic channel from left to right, as an example of cell counting experiments. The sequence has been cut to show the passing of three cells. This image sequence is acquired at 200 fps and slowed down 40 times.

Note S1: 3D design of the cell mixing device (*.stl)

Stl files of the 3D printed parts used for the assembly of the cell mixing device (CMD) shown in Figure 1 (see the main article). The files are available for direct printing.

Note S2: Image analysis procedure

The image analysis of cells flowing through the microchannel for cell counting experiments is performed by combining both specific tools of ImageJ software and using a homemade Labview programme as shown in the sequence in Figure S1. In detail, after the acquisition of the image sequence, a median filter is applied to reduce the contribution of electrical noise. Then, a background subtraction is performed to highlight the presence of the cells, and finally, a threshold filter is applied to get a binary image. Here, cells appear as white spots on a black background. Finally, the Labview algorithm allows to properly count the number of cells flowing through the microfluidic channel for each image sequence.

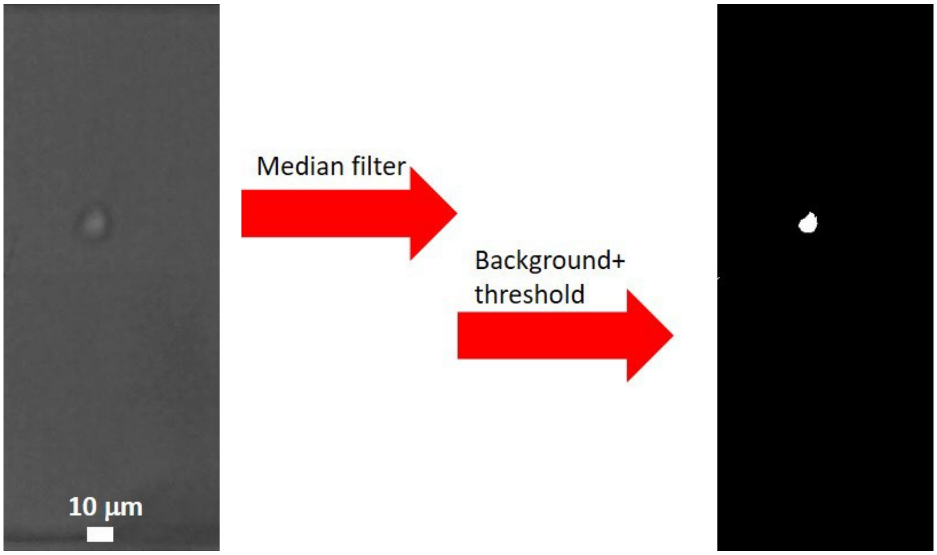

Supplementary Figure S1. Example of a raw image acquired during the experiment for cell counting and the same image in a post-processed version.

Note S3: Data acquired during cell counting experiments

Cell counting experiments are performed on different days with the procedure described in Section 3.3 (see the main article). For each experiment two kinds of configurations have been considered: with the CMD working (mixing) and without CMD (no mixing). Indipendent experiments have been performed on different days to ensure the reproducibility of the behaviour of CMD in limiting the sedimentation of cells. Here, in Figure S2a,b,c the data of the three experiments are reported as histograms, while Figure S2d shows the averaged data.

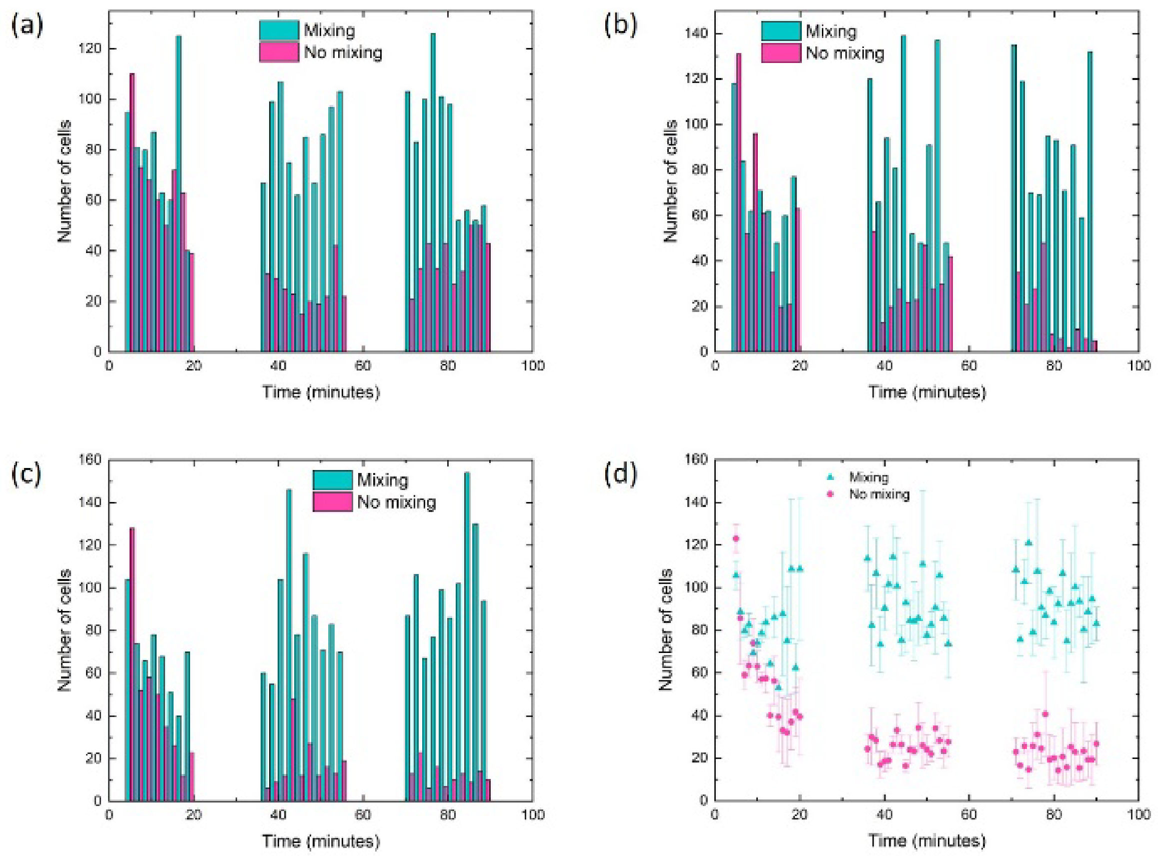

Supplementary Figure S2. (a), (b) and (c) Histograms showing the time distribution of the cell number in three different cell counting experiments under mixing and no mixing conditions. (d) Averaged data of the number of cells as a function of time collected from experiments carried out on different days (n=3). The cells come from the same cell culture, the oscillation frequency is 0.72 Hz (6 V) and cell concentration is 2 x 10^5 cells/ml.
